# Mental and Muscular Work in Relation to Food

**Published:** 1917-11-24

**Authors:** 


### MENTAL AND MUSCULAR WORK IN RELATION TO FOOD.

For many years the experimental physiologists have occupied themselves in an endeavour to teach their less scientific fellow-creatures what foods are best fitted for the supply of human needs and in what quantities and proportions these foods should be taken. The teachers have not always held a unanimous doctrine, and none of the schools has been able to boast anything like wholesale conversions or widespread triumph. On the contrary, the great majority of plain folk have followed their individual instincts, tastes, customs, or traditions, and have lived, more or less successfully, unredeemed by the pure truth of scientific dietetics. Nor has the so-called "food specialist," who at appropriate moments issues in the daily press his prophecies of woe, been more successful. He has his reward from the individual disciple, it is true, but the people generally remain unmoved by his threatenings. The terrors of "uric acid" and "oxalic acid" have commercial values among the credulous and the dyspeptic, but they do not disturb the food habits of the nation. For our own part we think there is a sound basis of wisdom in the popular choice, and against it we confidently expect that the percentage calculations of the laboratory and the dismal anticipations of the expert will continue to be urged in vain.

The events of the day, however, have brought us a new teacher, and his argument rests less upon chemical analysis than upon arithmetical calculation. He does not tempt us with visions of health and efficiency, but threatens us bluntly that, if we heed not his instruction, a worse thing will happen to us. The suggestion is that people should put up with a short supply in order to avoid the miseries of famine and the collapse of the national defence. There is no pretence that the frying-pan is a comfortable and desirable situation; the proposition merely is that it is better than the fire. It is quite possible that food restriction will be beneficial to a certain number of persons who, having nothing better to do, give excess of time and appetite to the pleasures of the table. But, with these exceptions, the restriction has a penal quality, and this aspect of the matter had better be frankly recognised. As one of our medical contemporaries has written, "the new scheme of rations is much less a hardship than actual famine," and with this severely chastened enthusiasm we fancy all will be able to agree. Necessity, not choice, is the sanction of the arrangement, and we must accept a limited ill in order to avoid a greater suffering. Some of the cranks have utilised the opportunity to promise untold blessings from the food shortage, but the immediate disadvantages are obvious enough, and the prophet nowadays is rather an exploded occupation. The short statement is that the national position is such that the individual citizen must learn to practise self-denial and endurance, and must be ready to make some sacrifice of personal comfort and even of personal efficiency. Talk about the benefits to health and well-being is merely a childish attempt to gild the pill, and is more likely to promote resentment than acquiescence. The plain truth is that for the majority of people a liberal supply of good food is the condition which best promotes both good health and vigorous working, and in so far as the supply is limited there will be a decline from these standards.

The new teacher has now proceeded to a further lesson. In the latest food proposals which he has put before the country as a scheme of voluntary rationing a large recognition is paid to the accepted creed that the sedentary worker needs a far less liberal supply of food than does the manual or industrial worker. We are not inclined to contest this as a general proposition, but it may be well to point out that the term "sedentary work" covers conditions which agree with one another only in a superficial and mechanical sense. At one extreme is, say, the clerk who has merely to take down what is dictated to him or to copy figures from an old book into a new one. At the other is the man who, under the pressure of large responsibilities, has to devise complicated plans and to take anxious and serious decisions, with all the risks which these entail; and between these two are numerous gradations in which the proportions of routine and creative work exist in varied measure. Yet all these, in the official scheme, come, even as they do in the popular judgment, under the term "sedentary" workers. We have no alternative grouping to propose, for obedience rather than criticism is just now the supreme virtue. All the same, we doubt whether the most suitable food conditions for these various workers are identical. That excess in food is generally a hindrance to good brain work may be allowed. But not less true is it that men who perform severe mental tasks usually find that appetite suggests a generous diet, and that such a diet is a condition of their best work. In this there may, perhaps, be some element of habit and a natural rebound from the strain and severity of mental effort to the ease and relaxation of material comfort. But a custom or habit which is widely spread, even if it involves a measure of harm, must possess some solid core of advantage. To believe otherwise is to accuse the race of unrelieved folly. Hence we should be reluctant to challenge the brain worker who puts in his plea for a liberal dietetic recognition. It is, we think, true that many such workers reach their highest levels only if this condition is satisfied. But in the circumstances of the day we may have to content ourselves with something short of the best. The question of the moment is less concerned with the mental adventures of the individual than with the existence of the nation. Hence the brain worker, like his industrial fellow-citizens, must accommodate himself to the situation and must give us the best of which he is capable in the circumstances. We admit he has a good practical plea for a more generous treatment. But the hour does not admit of fine distinctions, and in the meantime he must be numbered with the mere performer of routine. In due season we shall hope to see him at a more liberal table, and then we shall expect his highest flights and his most fruitful inspirations.

